# Safety and Effectiveness of Edoxaban in Atrial Fibrillation Patients in Routine Clinical Practice: One-Year Follow-Up from the Global Noninterventional ETNA-AF Program

**DOI:** 10.3390/jcm10040573

**Published:** 2021-02-03

**Authors:** Raffaele De Caterina, Young-Hoon Kim, Yukihiro Koretsune, Chun-Chieh Wang, Takeshi Yamashita, Cathy Chen, Paul-Egbert Reimitz, Martin Unverdorben, Paulus Kirchhof

**Affiliations:** 1 Division of Cardiology, Department of Surgical, Medical and Molecular Pathology and Critical Care Medicine, Pisa University Hospital and University of Pisa, Via Paradisa 2, 56124 Pisa, Italy; 2 Division of Cardiology, Korea University College of Medicine and Korea University Medical Center, 73 Goryeodae-ro Seongbuk-gu, Seoul 02841, Korea; yhkmd@unitel.co.kr; 3 National Hospital Organization Osaka National Hospital, Osaka 540-0006, Japan; koretsune.yukihiro.ry@mail.hosp.go.jp; 4 Division of Cardiology, Department of Internal Medicine, Chang Gung University and Chang Gung Memorial Hospital, Taoyuan 33305, Taiwan; chcwang@cgmh.org.tw; 5 Department of Cardiovascular Medicine, The Cardiovascular Institute, Tokyo 106-0031, Japan; yamt-tky@umin.ac.jp; 6 Daiichi Sankyo, Inc., 211 Mount Airy Road, Basking Ridge, NJ 07920, USA; cchen@dsi.com (C.C.); munverdorben@dsi.com (M.U.); 7 Daiichi Sankyo Europe, GmbH, Zielstattstraße 48, 81379 Munich, Germany; Paul-Egbert.Reimitz@daiichi-sankyo.eu; 8 Center for Cardiovascular Research, University of Birmingham and SWBH and UHB NHS Trusts, IBR 136, Wolfson Drive, Birmingham B15 2TT, UK; 9 Department of Cardiology, University Heart and Vascular Center Hamburg, Martinistraße 52, 20251 Hamburg, Germany; 10 German Center for Cardiovascular Sciences (DZHK), Partner Site Hamburg/Kiel/Lübeck, 20251 Hamburg, Germany

**Keywords:** atrial fibrillation, anticoagulation, oral anticoagulants, edoxaban, major bleeding, death, stroke prevention, non-vitamin K antagonist oral anticoagulant (NOAC)

## Abstract

Non-vitamin K antagonist oral anticoagulants such as edoxaban are the standard of care for stroke prevention in patients with atrial fibrillation (AF). The Global Edoxaban Treatment in routiNe clinical prActice (ETNA)-AF program integrates prospective, observational, noninterventional regional studies from Europe, Japan, and other Asian countries, collecting data on patient characteristics and clinical outcomes in unselected patients treated with edoxaban for stroke prevention in AF. Overall, 26,823 patients completed a 1-year follow-up and were treated with edoxaban; either 60 or 30 mg once daily. The majority (82.6%) of patients received the recommended doses according to the local label. At baseline, the median (interquartile range) age was 75 (68, 80) years, the CHA_2_DS_2_-VASc score was 3.0 (2.0, 4.0), and the hypertension, abnormal renal and liver function, stroke, bleeding, labile international normalized ratio, elderly, drugs, or alcohol (HAS-BLED) score was 2.0 (2.0, 3.0). At one year, there were 273 (1.12%/year) major bleeding events, including 75 (0.31%/year) intracranial hemorrhages and 140 (0.57%/year) major gastrointestinal (GI) bleeds. There were 214 ischemic strokes (0.87%/year). Mortality was 3.03%/year (745 deaths), and cardiovascular mortality accounted for 40% of all deaths (1.22%/year, 299 cardiovascular deaths). In conclusion, stroke, intracranial hemorrhage, and other major bleeding events were low in patients with AF treated with edoxaban in routine care. Even on anticoagulation, cardiovascular death remained common.

## 1. Introduction

The non-vitamin K antagonist oral anticoagulants (NOACs) apixaban, dabigatran, edoxaban, and rivaroxaban are recommended as the preferred therapy for stroke prevention in eligible patients with atrial fibrillation (AF) [1,2,3,4,5,6,7]. As a result, NOAC use in this indication has increased considerably during the last decade [8]. As a next step, and to complement scientific evidence from the randomized controlled trials, noninterventional studies with these NOACs are being conducted to investigate the safety and effectiveness of NOACs in routine clinical practice [9,10,11,12,13]. These multiple large-scale observational studies are necessary because each compound exhibits relevant differences in pharmacokinetic and pharmacodynamic properties that may affect clinical outcomes. Differences include protein binding, renal excretion, metabolism, and drug–drug interactions and may explain published differences in clinical outcomes [1,2,3,4,5,14]. Here, we report the 1-year event data from the routine clinical use of edoxaban for stroke prevention in patients with AF enrolled in the Global Edoxaban Treatment in routine cliNical prActice (ETNA)-AF program, which complements the results of the randomized controlled ENGAGE AF-TIMI 48 phase 3 study [3].

## 2. Materials and Methods

### 2.1. Design

The detailed study design of the ETNA-AF program was previously published [13]. Briefly, the Global ETNA-AF program integrates data from several prospective, observational, and noninterventional regional studies from Europe (Germany, Austria, Switzerland, Belgium, Italy, Spain, United Kingdom, Ireland, the Netherlands, and Portugal), Japan, and other Asian countries [13,15,16]. Participating sites included hospitals and outpatient clinics. Trial registrations are as follows: Europe (NCT02944019), Japan (UMIN000017011), and Korea/Taiwan (NCT02951039).

The responsible ethics committees and institutional review boards approved the ETNA-AF study protocols except for Japan, where such approval is not required for postmarketing surveillance studies in compliance with the Japanese Pharmaceutical Affairs Act.

### 2.2. Inclusion and Exclusion Criteria

Patients with AF treated with edoxaban for stroke prevention according to the local label were eligible. Patients in Japan were eligible only if they were receiving edoxaban for the first time to prevent ischemic stroke and systemic embolic events (SEEs). The only exclusion criteria were failure to provide written informed consent and simultaneous participation in an interventional study.

### 2.3. Assessments and Outcomes

Baseline information collected included demographics, vital signs, AF history and diagnosis, renal and hepatic parameters, bleeding history, and edoxaban therapy details. Medical history, clinical events, and descriptions were coded using the Medical Dictionary for Regulatory Activities. A patient was considered as having a medical history of heart failure if one of the following criteria was fulfilled: documented congestive heart failure (CHF) or, if CHF was not documented, then documentation of ischemic cardiomyopathy; ejection fraction <40%; frequent dyspnea (≥1/day) without chronic obstructive pulmonary disease and with documented severe valvular heart disease, coronary heart disease post-myocardial infarction (MI), valve replacement, or hypertension treated with ≥3 drugs.

The following events were systematically captured at 12 and 24 months after enrollment: stroke, SEE, bleeding, transient ischemic attack (TIA), heart failure (HF), MI, acute coronary syndrome, all-cause death, and cardiovascular (CV)-related death. Bleeding was characterized as major, clinically relevant non-major (CRNM), or minor in accordance with the International Society on Thrombosis and Hemostasis [17]. Clinical events were reported based on physician diagnosis and assessment per available guideline definitions.

Edoxaban is dosed at 60 mg once-daily in patients that do not exhibit renal impairment (creatinine clearance: 15–50 mL/min), weight ≤60 kg, or concomitant use of certain P-glycoprotein inhibitors, which leads to the administration of 30 mg once daily.

### 2.4. Statistical Analysis

Results are presented by summary statistics (*n*, mean, standard deviation; or median, lower quartile, and upper quartile) for numerical parameters and absolute and relative frequencies for categorical variables. Baseline data are presented as frequencies and/or as summary statistics. The number of patients with at least one clinical event is presented separately for each type of clinical event and the time-to-event data are presented as annualized rates (cases per 100 patient-years and displayed as %/year). Cumulative incidence functions were computed as 1-S(t), where S(t) is the Kaplan–Meier estimate of the survival function.

## 3. Results

### 3.1. Patient Population

Overall, 26,823 patients were enrolled in the Global ETNA-AF program and completed the 1-year follow-up. This included 13,092 (48.8%) patients from Europe, 11,054 (41.2%) from Japan, and 2677 (10.0%) from South Korea and Taiwan. During the 1-year follow-up, 3757/26,823 (14.0%) had permanently discontinued edoxaban therapy and 2828/26,823 (10.5%) had withdrawn from the study.

### 3.2. Edoxaban Treatment

Based on the 1-year follow-up analysis set, 14,348 (53.5%) received edoxaban 60 mg and 12,475 (46.5%) received edoxaban 30 mg. The median duration of edoxaban treatment in patients who completed the 1-year follow-up was 366 days (interquartile range (IQR) 363, 366) with no difference across the regions (Europe, 366 (IQR 366, 366); Japan, 366 (337,366); Korea and Taiwan, 366 (IQR, 351, 366)). Overall, 82.6% of patients received the recommended dose according to the local label.

### 3.3. Baseline Demographics and Clinical Characteristics

The baseline demographics and clinical characteristics are shown in Table 1. The median age was 75 years (IQR 68, 80), and most patients (58.2%) were male. The median CHA_2_DS_2_-VASc score was 3.0 (IQR 2.0, 4.0), and the median modified hypertension, abnormal renal and liver function, stroke, bleeding, labile international normalized ratio, elderly, drugs, or alcohol (HAS-BLED) score was 2.0 (IQR 2.0, 3.0). The majority of patients had paroxysmal AF (*n* = 13,305; 51.7%). Relative to patients from Europe, patients from East Asia (Japan, Korea, and Taiwan) had lower body weight (mean ± standard deviation (SD), Europe, 81.0 ± 17.3 kg; Japan, 60.0 ± 12.7 kg; Korea and Taiwan, 66.0 ± 12.0 kg), and lower body mass index (mean ± SD, Europe, 28.1 ± 5.1 kg/m^2^; Japan, 23.5 ± 3.8 kg/m^2^; Korea and Taiwan, 25.0 ± 3.7 kg/m^2^). Patients from East Asia and Europe had similar median (IQR) modified HAS-BLED scores (Japan, 2.0 (2.0, 3.0); Korea and Taiwan, 2.0 (2.0, 3.0); Europe, 2.0 (2.0, 3.0)).

The patient medical history is shown in Table 1. Some regional differences are notable. The percentage of patients with a history of ischemic stroke or HF was highest in Japan (17.9% and 27.1%, respectively). History of ischemic stroke was lowest in Europe (5.9%), and the history of HF was lowest in Korea and Taiwan (12.1%). Patients from Japan had the most prevalent history of bleeding, including major bleeding (2.5%), CRNM bleeding (1.2%), any gastrointestinal (GI) bleeding (1.6%), and intracranial hemorrhage (ICH; 2.3%), compared with Europe, Korea, and Taiwan. Patients from Europe had the highest burden of comorbidities, including hypertension (77.1%), MI (4.3%), peripheral artery disease (3.3%), and chronic obstructive pulmonary disease (COPD; 9.2%), compared with Japan and Korea/Taiwan, respectively: hypertension (72.0% and 71.3%), MI (3.8% and 1.5%), peripheral artery disease (1.6% and 0.8%), and COPD (0.7% and 4.9%).

### 3.4. One-Year Clinical Events

**Figure 1:**
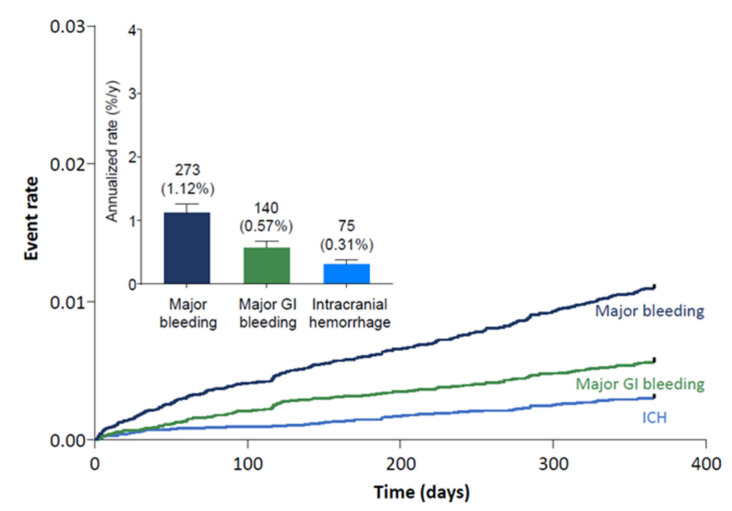
One-year major bleeding event rates. Numbers above bars represent n (%/y). Error bars represent the upper limit of the 95% confidence interval. GI, gastrointestinal; ICH, intracranial hemorrhage.

In the Global ETNA-AF population, bleeding event rates were low at the 1-year follow-up. Annualized event rates were 1.12% for major bleeding, 0.31% for intracranial hemorrhage, and 0.57% for major GI bleeding (Figure 1).

**Figure 2:**
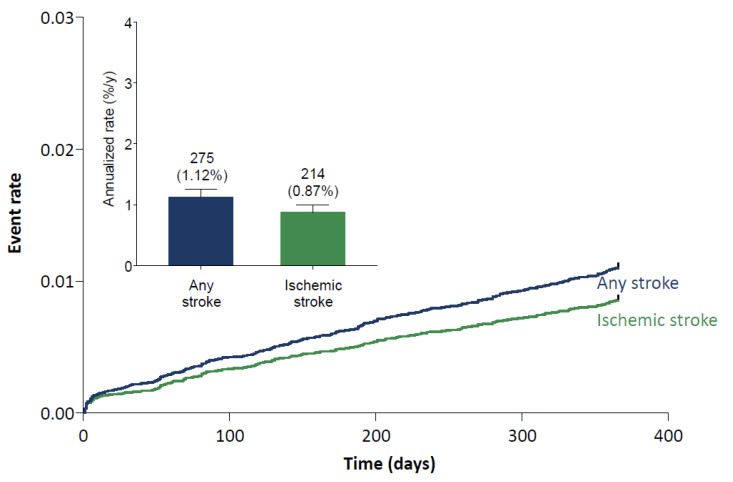
One-year stroke event rates. Numbers above bars represent n (%/y). Error bars represent the upper limit of the 95% confidence interval.

The rates of strokes and other thromboembolic events were low in the Global Edoxaban Treatment in routiNe clinical prActice (ETNA)-atrial fibrillation (AF) program. The 1-year annualized event rates were 1.12% for any stroke and 0.87% for ischemic stroke (Figure 2).

**Figure 3:**
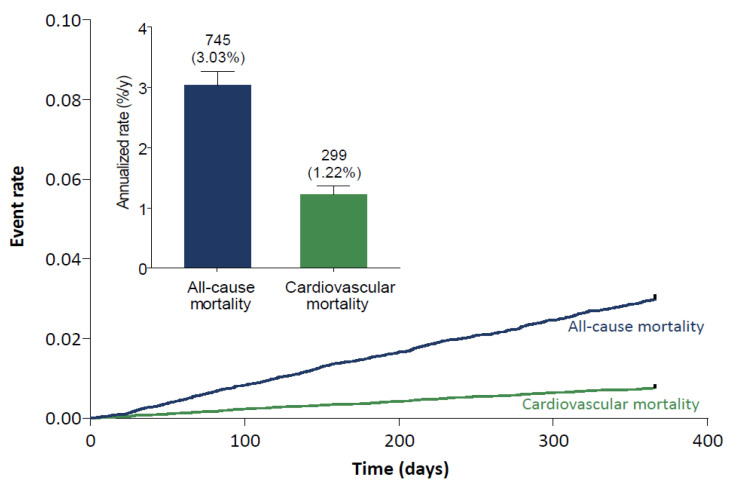
One-year mortality event rates. Numbers above bars represent n (%/y). Error bars represent the upper limit of the 95% confidence interval.

The rate of all-cause death was 3.03%/year (Figure 3). Regionally, all-cause death rates were highest in Europe (3.50%/year) and lowest in Korea and Taiwan (1.17%/year, Figure S1). Cardiovascular (CV) mortality in the global population was 1.22%/year (Figure 3). Regionally, rates were highest in Europe (1.63%/year) and lower in Japan (0.85%/year), Korea, and Taiwan (0.51%/year, Figure S1). In all regions, CV mortality remained a relevant contributor to mortality, even though stroke and bleeding were low.

## 4. Discussion

The Global ETNA-AF program is the largest prospective noninterventional program of a single NOAC. Here, we report the 1-year follow-up analysis from 26,823 patients with AF across European and Asian countries taking edoxaban 60- or 30-mg doses for the prevention of stroke. Overall, in this unselected, real-world population, the 1-year clinical event rates for stroke and major bleeding were low. Randomized controlled trials such as ENGAGE AF-TIMI 48 are global studies; however, patients in East Asian countries accounted for <10% of the ENGAGE population. In comparison, the Global ETNA-AF program has a larger proportion (~50%) from Japan, Korea, and Taiwan.

There were notable differences in patient demographics and clinical events across regions. All-cause mortality was lower in Korea and Taiwan (1.17%/year) compared with Europe (3.50%/year) and Japan (2.91%/year), possibly due to different baseline risk factors. Patients from Japan had the highest rates of heart failure as well as histories of major bleeding, ICH, and ischemic stroke compared with Europe and Korea/Taiwan. Despite these bleeding and stroke histories, the median modified HAS-BLED scores from Japanese and European patients were the same, and they had a similar mean age. The history of bleeding in Japanese patients may indicate a higher chance of bleeding events, including major bleeding, ICH, major GI bleeding, and CRNM bleeding observed in this study. Further, a systematic review and meta-analysis found that the incidence of ICH in Japan was about 2-fold higher than in patients of white ethnic origin [18]. Consistent with this finding, edoxaban-treated patients in Japan had an annualized ICH event rate at 1-year that was 1.7-fold higher than in European patients. While rates of major bleeding and ICH in South Korea and Taiwan were low (0.78% and 0.27%, respectively), rates of ischemic and hemorrhagic strokes were lower in patients from Europe (0.56% and 0.11%, respectively) compared with Japan (1.29% and 0.39%, respectively), and South Korea and Taiwan (0.90% and 0.19%, respectively). A clear causal relationship between baseline risk factors and differences in clinical events is not known, but it is interesting to speculate racial differences in the susceptibility to bleeding with a west-to-east gradient.

The 1-year major bleeding, CRNM bleeding, and stroke rates in the Global ETNA-AF compare favorably with the corresponding rates from the other smaller observational studies of edoxaban treatment for the prevention of stroke in AF. For instance, in a nationwide Danish registry of 2285 AF patients who received edoxaban over a 2-year observational period, the event rates per 100 person-years for ischemic stroke with the 60-mg and 30-mg doses were 1.51 and 1.74, respectively, and for major bleeding were 3.25 and 3.06, respectively [19]. In the ETNA-AF European population, the ischemic stroke and major bleeding rates were 0.56%/year and 1.05%/year, respectively. However, the Danish registry differs from ETNA-AF Europe in notable ways, including study design (Danish registry, retrospective vs. ETNA-AF, prospective), definition of bleeding, and patient populations, which should be considered when comparing the two registries [19].

The low rates of clinical events observed with edoxaban in patients from Korea and Taiwan in the Global ETNA-AF program compare favorably with the results of a survey of the Korean National Health Insurance Service database. Among 4200 AF patients in Korea taking edoxaban and 31,565 patients taking warfarin, edoxaban was associated with more favorable outcomes vs. warfarin with respect to ischemic stroke (3.22 events/100 person-years (PY) vs. 3.89 events/100 PY; hazard ratio (HR) 0.69), ICH (0.66/100 PY vs. 1.59/100 PY; HR 0.41), hospitalizations for GI bleeding (1.65/100 PY vs. 2.02/100 PY; HR 0.60), and hospitalizations for major bleeding (2.32/100 PY vs. 3.56/100 PY; HR 0.53) [20].

An effective anticoagulant for stroke prevention associated with low rates of major bleeding, particularly ICH, is especially valuable for patients from East Asia, as Asian ethnicity was reported to be a risk factor for ICH [21]. Despite the higher risk of ICH, Japanese patients have the lowest ICH-related case fatality (~17% vs. 42% global average at 30 days) [18].

Although it is difficult to directly compare the event rates in this real-world study to the results of the randomized controlled ENGAGE AF-TIMI 48 clinical trial, some findings are worth noting. The ischemic stroke and bleeding event rates with edoxaban in ETNA-AF were slightly lower than the corresponding rates from ENGAGE AF-TIMI 48 [3]. The event rate for ischemic stroke with edoxaban in ENGAGE AF-TIMI 48 was 1.25% vs. 0.87% in Global ETNA-AF [3]. Similarly, the annualized rates for major bleeding, major GI bleeding, and ICH in ENGAGE AF-TIMI 48 were 2.75%, 1.51%, and 0.39%, respectively [3]; whereas in the Global ETNA-AF population, the corresponding rates at 1-year were 1.12%, 0.57%, and 0.31%. ENGAGE AF-TIMI 48 enrolled a higher risk population as indicated by the CHADS2 score (2.8 in ENGAGE vs. 1.9 in ETNA-AF) and comorbidities, whereas the Global ETNA-AF program enrolled a broader range of patients, including those with a lower risk of ischemic stroke [13]. Patients in the Global ETNA-AF program were less likely than those in ENGAGE AF-TIMI 48 to have a history of HF (19.3% vs. 58.2%), hypertension (74.4% vs. 93.7%), diabetes (23.3% vs. 36.4%), and prior stroke or TIA (14.9% vs. 28.1%) [3]. Thus, the ETNA-AF population may be considered less sick than the ENGAGE population.

The Global ETNA-AF program could have benefited from a direct comparator arm. However, with the number of available oral anticoagulants (four NOACs plus warfarin/vitamin K antagonists (VKAs)), the total number of patients needed to be enrolled would have been unfeasible with an acceptable time frame. Therefore, no direct conclusions can be drawn from this study about the effectiveness or safety of edoxaban relative to VKAs or to other NOACs. We acknowledge that no conclusions in general about the efficacy (vs. “effectiveness”) of any NOAC vs. VKAs or between any of the NOACs can be drawn from non-randomized clinical studies.

Despite these limitations, the Global ETNA-AF program provides information on the use of edoxaban in AF patients in routine clinical care in the largest single NOAC registry conducted so far. This 1-year follow-up analysis reveals key demographic and clinical characteristics of AF patients who were prescribed edoxaban, and low event rates for stroke and bleeding were observed for a large global population with more than half of the patients from East Asia.

## Figures and Tables

**Table 1:** Baseline demographics and clinical characteristics.

	Global N = 26,823	Europe *n* = 13,092	Japan *n* = 11,054	Korea and Taiwan *n* = 2677
Age, median (IQR)	75 (68, 80)	75 (68, 80)	75 (68, 81)	72 (66, 78)
≥75 years of age, *n* (%)	13,519 (50.4)	6640 (50.7)	5796 (52.4)	1083 (40.5)
Male gender, *n* (%)	15,605 (58.2)	7430 (56.8)	6578 (59.5)	1597 (59.7)
Weight, kg, mean (SD)	70.8 (18.13)	81.0 (17.29)	60.0 (12.73)	66.0 (12.04)
BMI, kg/m^2^, mean (SD)	26.1 (5.02)	28.1 (5.11)	23.5 (3.81)	25.0 (3.70)
CrCl, mL/min, mean (SD) ^a^	68.7 (28.34)	74.3 (30.42)	63.9 (25.78)	63.3 (23.76)
Type of AF, *n* (%)				
Paroxysmal	13,305 (49.6)	7039 (53.9)	5103 (46.2)	1163 (43.6)
Persistent	8737 (32.6)	3481 (26.6)	4247 (38.4)	1007 (37.7)
Permanent	4744 (17.7)	2542 (19.5)	1702 (15.4)	500 (18.7)
Missing	37	30	2	7
CHA_2_DS_2_-VASc, median (IQR)	3.0 (2.0, 4.0)	3.0 (2.0, 4.0)	3.0 (2.0, 5.0)	3.0 (2.0, 4.0)
CHA_2_DS_2_-VASc, mean (SD)	3.2 (1.5)	3.1 (1.4)	3.5 (1.6)	3.0 (1.4)
HAS-BLED, median (IQR) ^b^	2.0 (2.0, 3.0)	2.0 (2.0, 3.0)	2.0 (2.0, 3.0)	2.0 (2.0, 3.0)
HAS-BLED, mean (SD) ^b^	2.4 (1.1)	2.5 (1.1)	2.4 (1.1)	2.2 (1.0)
Previous VKA, *n* (%)	4110 (15.3)	2262 (17.3)	1408 (12.7)	440 (16.4)
Previous NOAC, *n* (%)	2978 (11.1)	1062 (8.1)	1218 (11.0)	698 (26.1)
Medical history, *n* (%)				
Ischemic stroke	3147 (11.7)	778 (5.9)	1980 (17.9)	389 (14.5)
Transient ischemic attack	845 (3.2)	448 (3.4)	345 (3.1)	52 (1.9)
Major bleeding	464 (1.7)	129 (1.0)	279 (2.5)	56 (2.1)
CRNM bleeding	301 (1.1)	144 (1.1)	135 (1.2)	22 (0.8)
Intracranial hemorrhage	357 (1.3)	62 (0.5)	257 (2.3)	38 (1.4)
Any GI bleeding	365 (1.4)	163 (1.2)	175 (1.6)	27 (1.0)
Comorbidities, *n* (%)				
Hypertension	19,952 (74.4)	10,088 (77.1)	7954 (72.0)	1910 (71.3)
Diabetes mellitus	6241 (23.3)	2879 (22.0)	2574 (23.3)	788 (29.4)
Myocardial infarction	1019 (3.8)	560 (4.3)	420 (3.8)	39 (1.5)
Heart Failure	5175 (19.3)	1854 (14.2) ^c^	2997 (27.1)	324 (12.1)
Peripheral artery disease	632 (2.4)	437 (3.3)	174 (1.6)	21 (0.8)
Chronic obstructive pulmonary disease	1415 (5.3)	1206 (9.2)	77 (0.7)	132 (4.9)
Hepatic disease	1125 (4.2)	215 (1.6)	818 (7.4)	92 (3.4)

^a^ Calculated using the Cockcroft–Gault equation. ^b^ The HAS-BLED score has been calculated without the term “labile INR”. ^c^ A new definition of heart failure was applied to the data, which differs from the congestive heart failure value in reference [15]. AF, atrial fibrillation; BMI, body mass index; CHA_2_DS_2_-VASc, CHF, Hypertension, Age (≥65 = 1 point, ≥75 = 2 points), Diabetes, Prior Stroke/Transient Ischemic Attack (2 points), Vascular disease, and Sex category; CrCL, creatinine clearance; CRNM, clinically relevant non-major; NOAC, non-vitamin K antagonist oral anticoagulant; GI, gastrointestinal; HAS-BLED, hypertension, abnormal renal and liver function, stroke, bleeding, labile international normalized ratio, elderly, drugs or alcohol; IQR, interquartile range; N/A, not available; SD, standard deviation; VKA, vitamin K antagonist.

## Data Availability

Data will not be made available from this analysis because the study is still ongoing.

## Supplementary Materials

The following are available online at https://www.mdpi.com/2077-0383/10/4/573/s1, Figure S1: Annualized mortality by region.
