# Supplementary material for: Safety and Effectiveness of Edoxaban in Atrial Fibrillation Patients in Routine Clinical Practice: One-Year Follow-Up from the Global Noninterventional ETNA-AF Program
Source: J Clin Med. 2021 Feb 3;10(4):573. doi: 10.3390/jcm10040573 (PMC7913627; doi:10.3390/jcm10040573)
Supplement: Supplementary file 1 [file jcm-10-00573-s001.pdf]

Supplemental data

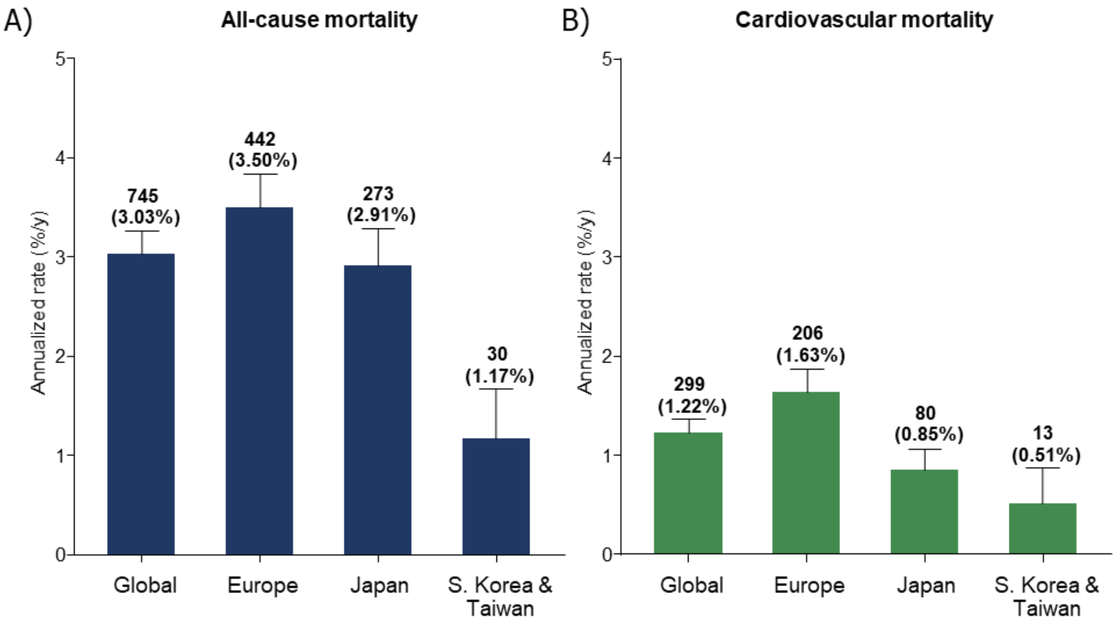

Figure S1. Annualized mortality by region. Numbers above bars represent n (%/y). Error bars represent the upper limit of the 95% confidence interval.
